# Hyperbaric oxygen suppressed tumor progression through the improvement of tumor hypoxia and induction of tumor apoptosis in A549-cell-transferred lung cancer

**DOI:** 10.1038/s41598-021-91454-2

**Published:** 2021-06-08

**Authors:** Shao-Yuan Chen, Koichi Tsuneyama, Mao-Hsiung Yen, Jiunn-Tay Lee, Jiun-Liang Chen, Shih-Ming Huang

**Affiliations:** 1 Department of Hyperbaric Medicine and Neurology, Cardinal Tien Hospital, New Taipei City, Taiwan, ROC; 2 School of Medicine, Fu Jen Catholic University, New Taipei City, Taiwan, ROC; 3 Graduate Institute of Aerospace and Undersea Medicine, National Defense Medical Center, Taipei, Taiwan, ROC; 4 Department of Molecular and Environmental Pathology, The University of Tokushima, Tokushima, Japan; 5 Department of Pharmacology, National Defense Medical Center, Taipei, Taiwan, ROC; 6 Department of Neurology, Tri-Service General Hospital, National Defense Medical Center, Taipei, Taiwan, ROC; 7 Department of Traditional Chinese Medicine, Chang Gung University, Taoyuan City, Taiwan, ROC; 8 Department of Biochemistry, National Defense Medical Center, Taipei, Taiwan, ROC

**Keywords:** Cancer, Diseases

## Abstract

Tumor cells have long been recognized as a relative contraindication to hyperbaric oxygen treatment (HBOT) since HBOT might enhance progressive cancer growth. However, in an oxygen deficit condition, tumor cells are more progressive and can be metastatic. HBOT increasing in oxygen partial pressure may benefit tumor suppression. In this study, we investigated the effects of HBOT on solid tumors, such as lung cancer. Non-small cell human lung carcinoma A549-cell-transferred severe combined immunodeficiency mice (SCID) mice were selected as an in vivo model to detect the potential mechanism of HBOT in lung tumors. HBOT not only improved tumor hypoxia but also suppressed tumor growth in murine xenograft tumor models. Platelet endothelial cell adhesion molecule (PECAM-1/CD31) was significantly increased after HBOT. Immunostaining of cleaved caspase-3 was demonstrated and apoptotic tumor cells with nuclear debris were aggregated starting on the 14th-day after HBOT. In vitro*,* HBOT suppressed the growth of A549 cells in a time-dependent manner and immediately downregulated the expression of p53 protein after HBOT in A549 cells. Furthermore, HBOT-reduced p53 protein could be rescued by a proteasome degradation inhibitor, but not an autophagy inhibitor in A549 cells. Our results demonstrated that HBOT improved tissue angiogenesis, tumor hypoxia and increased tumor apoptosis to lung cancer cells in murine xenograft tumor models, through modifying the tumor hypoxic microenvironment. HBOT will merit further cancer therapy as an adjuvant treatment for solid tumors, such as lung cancer.

## Introduction

Tumor hypoxia has been a concern for cancer therapy^1,2^. Hypoxia can increase tumor resistance to chemotherapy, radiation and lead to malignant progression and even metastasis^3,4^. Hypoxia or some signaling alterations of the hypoxic cascades could enhance the intact neovascularization, and facilitate tumor metastasis^5,6^.

Hyperbaric Oxygen Treatment (HBOT) is a medical treatment using 100% oxygen administered at a greater than usual atmospheric pressure. Since cellular and vascular proliferation is promoted by HBOT in an ischemic wound^7,8^, practitioners of hyperbaric medicine are concerned about the effect of HBOT on cancer growth^9^. However, more and more evidence suggests a neutral effect of HBOT on malignancy^10–12^. HBOT working as an adjuvant to promote cellular and vascular proliferation might have the same effect in a tumor. However, the nature physiology between tumor growth and wound healing differs by way of cancer growth, transformation and metastases. Such differences will result in different impacts on HBOT. A significant difference pointed out by Growther et al.^13^ suggested macrophages are the major healing factors in angiogenesis, while tumor macrophages contributed as part as an angiogenesis factor in a tumor environment^14,15^. The optimal oxygen tensions of 30 to 40 mmHg increased by HBOT can stimulate collagen synthesis and hydroxylation in wounds, but it does not promote cell proliferation in cancer.

Our previous experiments demonstrated that exposure to HBO attenuated the severity of disease progression in autoimmune NZB/W F1 mice^16^, proposing targeted apoptosis to hyper-proliferating lymphocytes. Moreover, hematopoietic origin cells have a lower threshold to oxidative stress induced by HBOT and HBO-induced apoptosis of hematopoietic-derived cancer cells was through the intracellular accumulation of H_2_O_2_ and O_2_^**.-**^ as well as the involvement of phosphorylation of p38 MAPK^17^.

Lung cancer is responsible for more than one million deaths annually and is claimed to be the world's most popular cancer. The main types of lung cancer are small-cell lung cancer (SCLC), and non-small-cell lung cancer (NSCLC). Several studies so far have provided direct beneficial shreds of evidence to show the effective combined therapy with HBOT and chemotherapy for cancer^18–21^. The reasons for the successfully combined therapy are the good vascularization of hypoxic tumor cells after HBOT. Hypoxic tumor cells are much more progressive and have the potentials to be metastatic. Moreover, tumor cells under hypoxic situations are more resistant to chemotherapy. In several clinical studies, combined HBOT and chemotherapy demonstrated practical value of increased survival rate and the side effects did not increase by combining HBOT with chemotherapy^22–24^.

Our study aimed to establish human lung carcinoma A549 tumor-transferred severe combined immunodeficiency mice (SCID) mice to investigate the potential role of HBOT in cancer therapy. NSCLC A549-transferred SCID mice were selected to detect the long-term beneficial effects of HBOT in cancer therapy without any combined treatment in the manner of 2.5 ATA/90 min/day in 2 weeks after 45 days of tumor transfer. Tumor hypoxia detected by Hypoxyprobe-1 was examined and the effect of HBOT on tumor growth was demonstrated after 14 and 28 days of HBOT. Further, the direct effects of HBOT on cell base, including cell viability and related protein expressions (such as p53 and HIF-1) were detected in A549 cells. Hence, this study will be the first experiment to provide both in vivo and in vitro responses of tumor cells to HBOT and propose the underlined tumor microenvironment for further solid cancer treatment.

## Materials and methods

### Cell culture

Human lung carcinoma A549 cell line and an immortalized human bronchial epithelial cell line, Beas-2B, were purchased from the Food Industry Research and Development Institute (Hsinchu, Taiwan, R.O.C.). The cells were cultured at 10^6^ cells/ml in RPMI 1640 with 10% fetal calf serum, 2 mM L-glutamine, 1 mM sodium pyruvate, 4.5 g/l glucose, 10 mM HEPES, and 1.5 g/l sodium bicarbonate. Individual cultures were maintained for no more than two months.

### Mice

50 C.B-17 SCID female mice were purchased from National Taiwan Medical Animal Center and maintained in a pathogen-free and, individually ventilated cage sat National Defense Medical Center- Laboratory Animal Center (NDMCLAC), Taiwan. Routinely sterilized, rodent diets (Meika) were used to feed the mice. The mice were used for the experiments at the ages of 6 to 8 week. All animal experiments were in accordance with NDMCLAC guidelines of IACUC. The animal protocols were approved by the IACUC in Taiwan (IACUC-101A01-002, IACUC-13–264). The study was carried out in compliance with the ARRIVE guidelines.

### Injection of A549 tumors

Mice were anesthetized by intra-peritoneum (i.p.) injection of pentobarbital: 40 mg/kg body weight. The abdomen was sterilized with 2-isopropanol (70%). A549 cells were diluted to 6 × 10^6^ cells in a volume of 0.2 ml RPMI 1640 and transferred to each recipient at the neck site by subcutaneous injection. The diameter of tumor mass was monitored two times per week and only mice with tumor volume larger than 500mm^3^ were selected for further experiments.

### Hyperbaric oxygen treatment (HBOT)

A549-tumor transferred SCID mice were either non-exposed or exposed to HBO in a hyperbaric animal chamber (98% O_2_, 2% CO_2_ at 2.5 ATA; 0.5 ATA/min to a pressure of 2.5 ATA) (Longshin Gas Ltd. Taipei, Taiwan, R.O.C.) for 90 min once a day over two weeks beginning on the 45th date after tumor injection. Cells in groups were exposed to HBOT (98% O2, 2% CO2 at 3.5 ATA) (Longshin Gas Ltd., Taipei, Taiwan, R.O.C.) in a hyperbaric chamber for 90 min. Control cultures for each experiment were placed in an incubator at 37 ^◦^C, 21% O2, 5% CO2 at 1 ATA. This hyperbaric chamber was maintained at a concentration of 95% oxygen, and CO_2_ was exhausted at a rate of 10–12 L/min. After reaching the end of the treatment, the chamber was slowly decompressed at 0.5 ATA/min pressure.

### Tumor volume

We measured tumor volume two times in a week and calculated the tumor size as length (L) × width (W) × height (H).

### Detection of tumor hypoxia

Hypoxyprobe-1 kit (Pimonidazole Hydrochloride) (Chemicon International) was used to measure tissue hypoxia in solid tumors. A dose of 60 mg/kg body weight of Hypoxyprobe-1 kit was injected intraperitoneally to A549-transferred SCID mice one hour before they were sacrificed. Tumor mass was removed and embedded as OCT/liquid nitrogen cryostat section.

### Tumor histopathology

Tumor cells were removed from mice for either frozen or paraffin sections. For frozen sections, tissue cells were snap-frozen in dry ice-cold 2-methylbutane and embedded in TISSUE-TEC (Miles, Elkhart, IN, U.S.A.). Freshly cut Sects. (5 μm) were mounted on clean glass slides coated with poly-L-lysine (Sigma Chemical Co, U.S.A.). They were further rapidly air-dried and stored at − 80 °C until used for immunohistochemical staining. The fixed sections were incubated with normal goat serum diluted 1:5 in PBS for 15 min at room temperature to block nonspecific staining. Sections were incubated with primary antibodies including goat anti-mouse CD31 antibody (BD Pharmingen, San Diego, CA, U.S.A.), rabbit anti-mouse cleaved caspase-3 antibody (BD Pharmingen, San Diego, CA, U.S.A.) and rabbit anti mouse VEGF antibody (BD Pharmingen, San Diego, CA, U.S.A.) in PBS for 1 h at room temperature in a moist chamber and then washed with PBS for 5 min with gentle shaking. After washing in PBS, they were interacted with immunopolymer of anti-goat or rabbit IgG antibodies (Histofine, Nichirei, Japan and Dako) as secondary antibodies and developed with DAB chromogen (Vector) for 6 min. The slides were washed with Q water and incubated with hemotaxylin for staining nucleus. The sections were immersed with 75%–95%–100% alcohol and xylene before covered with a coverslip. The processing, embedding and sectioning of paraffin blocks were performed in Dr. Koichi Tsuneyama’s pathology laboratory and the paraffin sections were deparaffinized and re-hydrated before immunohistochemical staining. To evaluate the immunostaining results objectively, whole slide scanning with computer counting was performed. The slides were scanned with slide scanner (3DHISTECH, Budapest, Hungary) in 200X magnification. Computer counting software of CellQuant and PatternQuant were used (3DHISTECH, Budapest, Hungary). Briefly, PatternQuant was trained for recognizing region of interests and following CellQuant to evaluate the H-Score. H-score was defined by immuno intensity multiply by staining percentage (range from 0 to 300). Immuno intensity was recorded as 0 for no staining, 1 for faint staining, 2 for moderate staining, and 3 for intense staining. The staining percentage was recorded from 0 to 100%. Both immuno intensity and staining percentage were automatically calculated by CellQuant which would only count on region of interests recognized by PatternQuant.

### Western blotting

Cell lysates were prepared in lysis buffer (100 mM Tris–HCl of pH 8.0, 150 mM NaCl, 0.1% SDS, and 1% Triton X-100) at 4 °C. The cell extracts were boiled and resolved on 10% SDS-PAGE gels. After the protein was transferred to nitrocellulose membranes by electroblotting (Bio-Rad, Richmond, CA, U.S.A.), the blots were blocked by overnight incubation with 5% nonfat dry milk in Tris/boric acid/sodium chloride/Tween20 and subsequently probed with antibodies againstp53, ACTN, and Nrf-2 (Santa Cruz, U.S.A.), LC3B, HIF-1α, PARP, Caspase-3, and p-p53 (Ser15) (Cell Signaling, U.S.A.), and γ-H2A.x (Abcam, U.K.). Immunoreactive proteins were visualized using horseradish peroxidase-linked secondary antibodies and further with ECL (Enhanced-chemiluminescence western blotting kit) (Amersham Biosciences, Pittsburgh, PA, U.S.A).

### Reverse transcription-polymerase chain reaction (RT-PCR)

Total RNA was isolated using TRIzol (Thermo Fisher Scientific, U.S.A.) reagent according to the manufacturer’s instructions. For 1st-strand cDNA synthesis, 1 µg of total RNA was reverse transcribed using 200 U of M-MLV reverse transcriptase for 60 min at 37 °C (Epicentre Biotechnologies, U.S.A.). Specific primers (for p53: Forward: 5′-CTCTGACTGTACCACCATCCACTA-3′; Reverse: 5′-GAGTTCCAAGGCCTCATTCAGCTC-3′; for GAPDH: Forward: 5′-CTT CAT TGA CCT CAA CTA C-3′; Reverse: 5′-GCC ATC CAC AGT CTT CTG-3′), dNTP, and Taq DNA polymerase were added for subsequent PCR reactions, which were processed using a Veriti Simpli Thermal Cycler (Applied Biosystems, CA, U.S.A.).

### Statistical analysis

Unpaired t-test, one-way ANOVA and Nonparametric Kruskal–Wallis test were used to compare differences between mean*s* of groups and statistical analysis was performed using SPSS. One-Dscan analysis software was used to analyze the expression contents of proteins in western blotting.

### Ethical approval and consent to participate

All animal experiments were in accordance with NDMCLAC guidelines of IACUC. All animal protocols were approved by the IACUC in Taiwan (IACUC-101A01-002, IACUC-13-264).

## Results

### HBOT suppressed tumor growth

**Figure 1:**
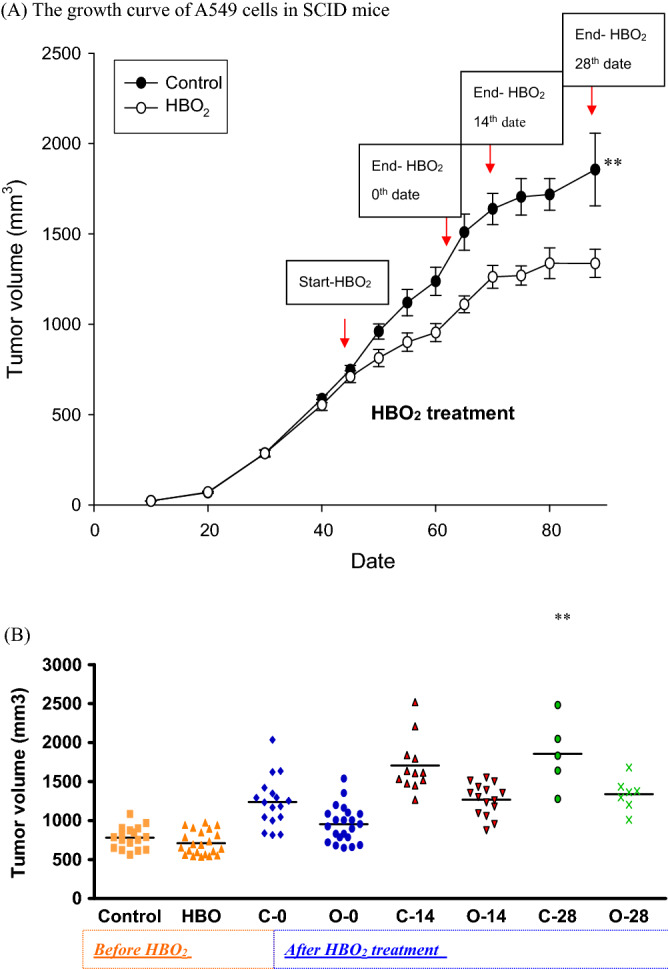
A549-tumor transferred SCID mice were either non-exposed or exposed to HBO_2_(98% O_2_, 2% CO_2_ at 2.5 ATAfor2.5 ATA 90 min 5 times in a week beginning at the 45th date after tumor injection and were monitored for the tumor volume twice in a week. Tumor volume was calculated as length (L) × width (W) × height (H). (**A**) The growth curve of A549 cells in SCID mice. Only the mice with tumor volume larger than 500mm^3^ were selected (**B**) The comparison of tumor volume among groups of mice before or after HBOT. For each dot-point represents the tumor volume of one A549-tumor transferred SCID mice. HBOT effectively suppressed tumor growth after 14 days (**P < 0.01) and 28 days (**P < 0.01) of tumor transfer. (C-0 represents for control mice at the first date comparing with HBOT mice; O-0 represents for mice receiving the first date of HBOT; C-14 days represents for control mice after 14 days comparing with HBOT mice; O-14 days represents for mice after 14 days of HBOT; C-28 days represents for control mice after 28 days comparing with HBOT mice; O-28 days represents for mice after 28 days of HBOT).

On the observation that HBOT could decrease the proliferation rate of A549 cells^17^, we established the A549 tumor transferred-SCID mice xenograft model for the potential use of HBOT (2.5 ATA, 90 min) in cancer therapy. A549-tumor transferred SCID mice were either non-exposed or exposed to HBO for 2.5 ATA 90 min 5 times in a week beginning on the 45th date after tumor injection and were monitored for the tumor volume twice in a week. The tumor volume of human lung carcinoma A549 cells was increased at a linear curve after 20 days of transfer for both control and HBO-treated mice (Fig. 1A). Only the mice with tumor volume larger than 500mm^3^ were selected to HBOT. Our data showed that HBOT effectively suppressed tumor growth after 14 days (**P < 0.01) and 28 days (**P < 0.01) of tumor transfer (Fig. 1A,B) compared with control mice.

### HBOT improved tumor hypoxia

**Figure 2:**
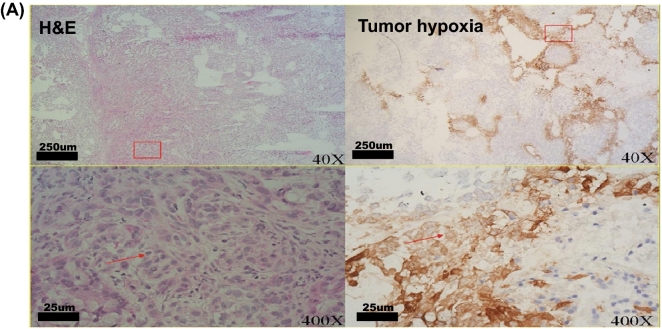

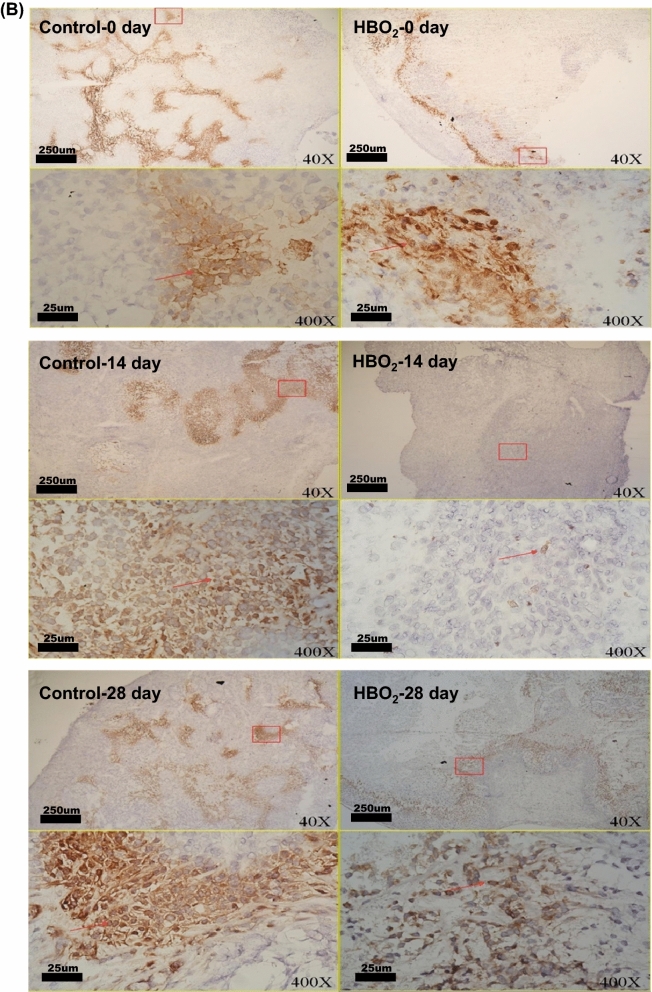
Immunohistochemical staining of tumor sections from A549- transferred SCID mice. (**A**) Haematoxylin & Eosin (H & E) staining and immunostaining of Hypoxyprobe-1 probe of A549 tumor frozen sections after 45 days of tumor transfer. (H & E: H and E staining; tumor hypoxia: Immunostaining of Hypoxyprobe-1 probe) (**B**) Comparisons of tumor hypoxia in groups of mice after 14 and 28 days of HBOT. (Control-0: control mice at the first date comparing with HBOT mice; HBO_2_-0: mice receiving the first date of HBOT; Control-14: control mice after 14 days comparing with HBOT mice; HBO_2_-14: mice after 14 days of HBOT; Control-28: control mice after 28 days comparing with HBOT mice; HBO_2_-28: mice after 28 days of HBOT).

Although tumor sections from A549-transferred SCID mice showed the regular shapes after 45 days of tumor transfer by hematoxylin& eosin (H & E) staining, significant tumor hypoxia was measured by Hypoxyprobe-1 probe (Fig. 2A). HBOT significantly improved tumor hypoxia after 14 days (*P < 0.05) and 28 days (*P < 0.05) of HBO therapy (Fig. 2B, Figure S3 and Table 1) by quantitative analysis.

**Table 1:** Semi-quantitative expressions of tumor hypoxia, CD31 and VEGF in groups of mice after 14 and 28 days of HBOT.

	Control-14 (N = 10)	HBO_2_-14 (N = 10)	Control-28 (N = 10)	HBO_2_-28 (N = 10)
Tumor hypoxia (mean ± SD)	3.40 ± 0.55	*1.25 ± 0.96	3.20 ± 0.37	*2.00 ± 0.01
CD31 (mean ± SD)	2.20 ± 0.45	*3.25 ± 0.50	2.00 ± 0.32	*2.25 ± 0.48
VEGF (mean ± SD)	1.40 ± 0.55	1.25 ± 0.50	1.20 ± 0.20	1.25 ± 0.25

1. Cell staining	0%	No staining	0	
	0–1%	Nuclear staining in less than 1% of cells	1	
	1–10%	In 1–10% of cells	2	
	10–50%	In 10–50% of cells	3	
	> 50%	Greater than 50%	4	
2. Nonparametric Kruskal–Wallis test				
3. *P < 0.05, compared with Control group				

### HBOT induced apoptosis in A549 tumor transferred SCID mice.

**Figure 3:**
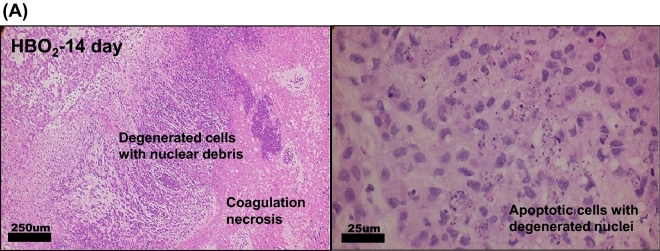

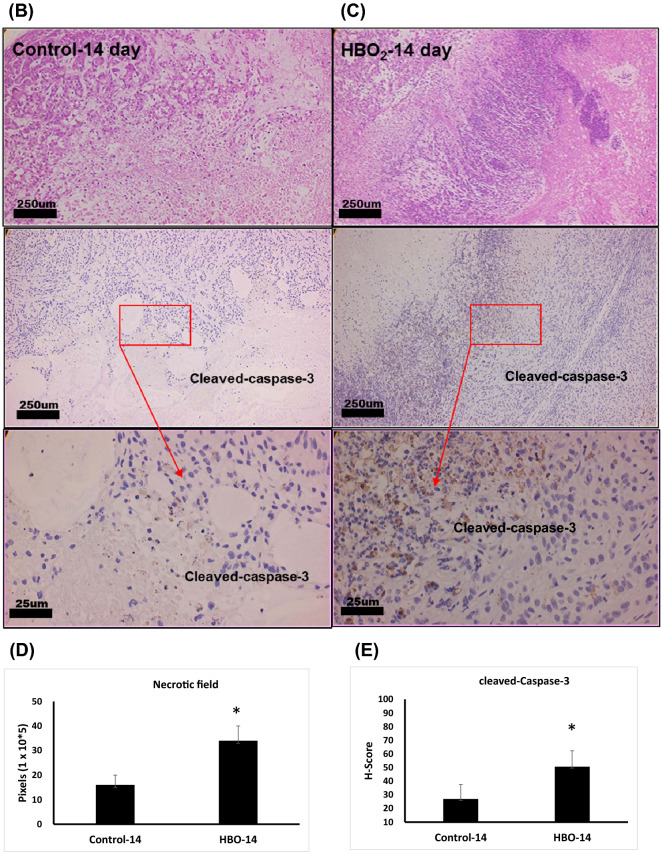
Immunohistochemistry staining of tumor sections from A549- transferred SCID mice. (**A**) Tumor paraffin sections of HBO_2_-14th date showed zonal inflammatory cells and degenerated cells aggregation in the border of coagulation necrosis. Immunostaining of cleaved-caspase-3 after 14 days of HBOT in HBO_2_ group (**B**) and control group (**C**). Note that, in HBOT group, many cleaved-caspase-3 positive apoptotic cells were located in the border between coagulation necrosis and viable tumor cells. Comparative analysis of necrotic field (**D**) and cleaved-caspase-3 (**E**) was quantified from groups of mice. Statistical significance (*P < 0.05, compared with control group) was observed in HBOT group. H-score was defined by immuno intensity multiply by staining percentage (range from 0 to 300). Computer counting software of CellQuant and PatternQuant were used (3DHISTECH, Budapest, Hungary) for quantitation.

Histopathological findings of tumor lesions were quite similar on the first day after HBO exposure between control and HBOT groups. The necrotic field area is not observed on the first day after HBOT; however, the necrotic field was more prominent on the 14th and 28th date after HBOT (Fig. 3D). Ghost-like shadow of cells was recognized in the necrotic field as “coagulation necrosis”. Tumor sections of HBOT-14th and 28th date showed zonal inflammatory cells and degenerated cells with nuclear debris were aggregated in the border of coagulation necrosis (Fig. 3A). Immunostaining of cleaved-caspase-3 was demonstrated in HBO-treated mice after 14 days of HBOT (Fig. 3B,E) compared with control mice (Fig. 3C,E).

### CD31 was significantly increased after HBOT.

**Figure 4:**
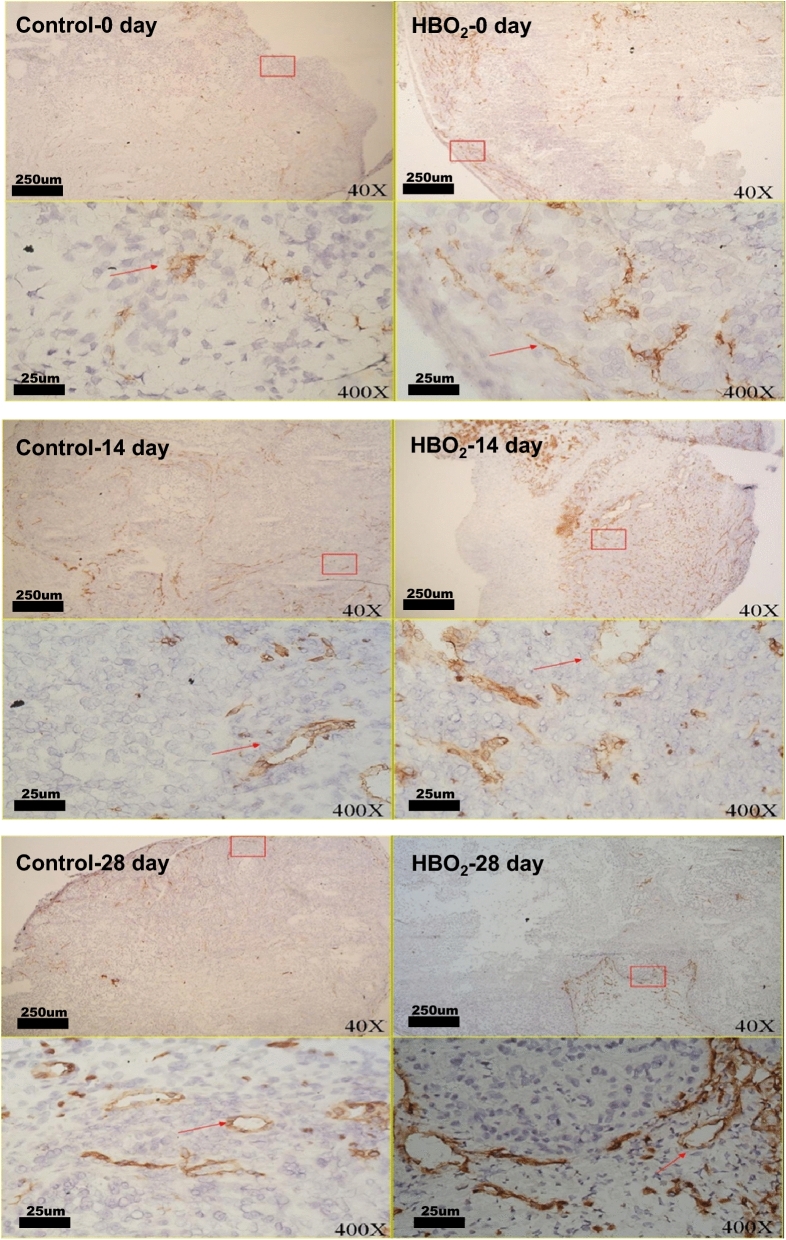
Comparative analysis of immunohistochemical CD31 antigen expressions from groups of mice. Significantly increase of CD31 was observed after 14 days and 28 days of HBOT exposure.

Platelet endothelial cell adhesion molecule (PECAM-1/CD31), is a ligand for CD38 and plays a role in angiogenesis. CD31 is highly expressed in endothelial cells and the expressions of vascular endothelial growth factor (VEGF) and CD31 have recently been implicated in tumor angiogenesis. HBOT did not change the expression of VEGF but significantly increased the expression of CD31 after 14 days and 28 days of HBOT (*P < 0.05) (Fig. 4, Figure S3 and Table 1).

### HBOT decreased p53 protein and increased HIF-1α protein in A549 cells, not Beas-2B cells.

**Figure 5:**
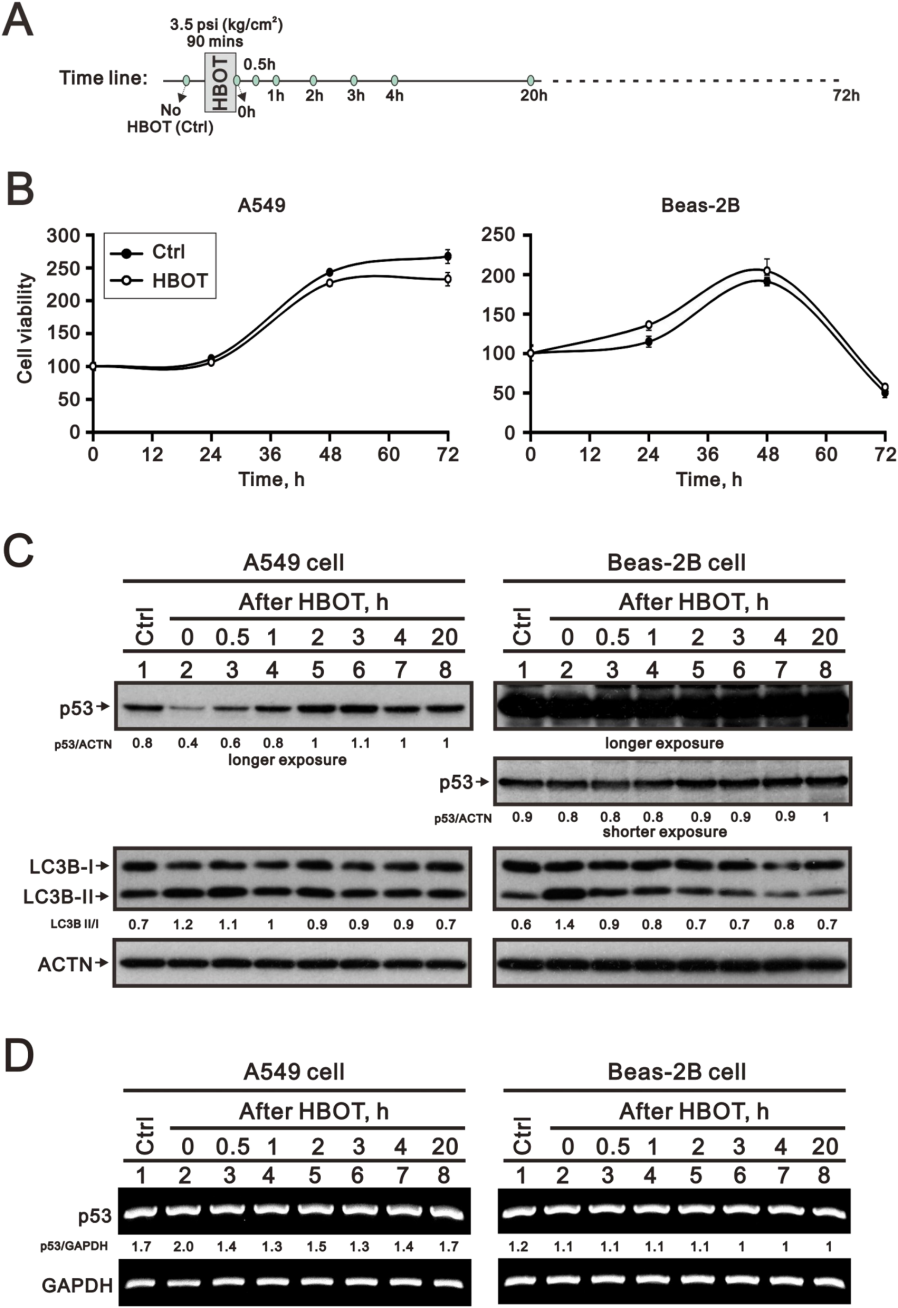
The HBOT protocol and its transient effect on A549 and Beas-2 cell lines. (**A**) The HBOT protocol was applied in A549 and Beas-2 cell lines. (**B**) The cell viability profiles were analyzed in A549 and Beas-2 cell lines. (**C**,**D**) The HBOT A549 and Beas-2 cells were treated with indicated conditions and then cell lysates were subject to the (**C**) Western analysis for antibodies against p53, LC3B, and control protein ACTN and (**D**) RT-PCR analysis for p53 mRNA and control GAPDH mRNA. The results were representative of two independent experiments. Quantitative analysis of the western blot was listed under each strip.

We examined the effects of HBOT on A549 cells compared with an immortalized human bronchial epithelial cell line, Beas-2B, followed by the cell-based HBOT protocol (Fig. 5A). HBOT suppressed the growth of A549 cells, not Beas-2B cells, in a time-dependent manner as measured in Fig. 5B. From western blotting analysis, p53 proteins in A549 cells were first downregulated by HBOT and then rebounded to basal level after 6 h of HBOT, but not Beas-2B cells (Fig. 5C). There is no significant difference in the expression of p53 mRNA after HBOT between A549 and Beas-2B cells (Fig. 5D).The LC3BII/I ratio was increased by the HBOT and then declined to basal level after HBOT in Beas-2B cells (Fig. 5C, Figure S1).

### A proteasome degradation inhibitor could rescue HBOT-reduced p53 protein, but not an autophagy inhibitor in A549 cells

**Figure 6:**
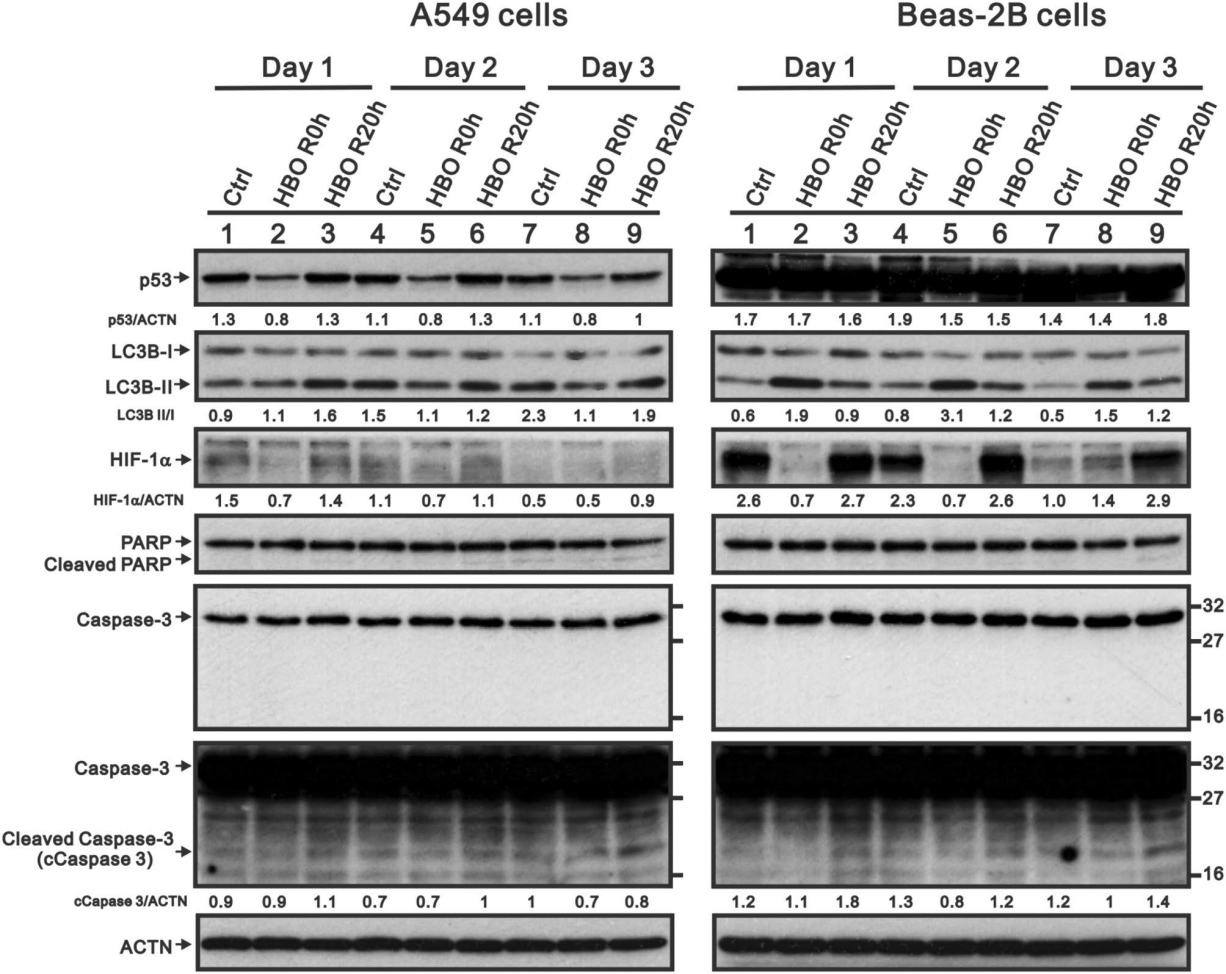
The HBOT effect on stress proteins on A549 and Beas-2 cell lines. The A549 and Beas-2 cells were treated with HBOT at 3.5 ATA for 90 min and then lysates were subject to the Western analysis for antibodies against p53, LC3B, HIF-1α, PARP, Caspase 3, and control protein ACTN. The results are representative of two independent experiments. Quantitative analysis of the western blot was listed under each strip.

**Figure 7:**
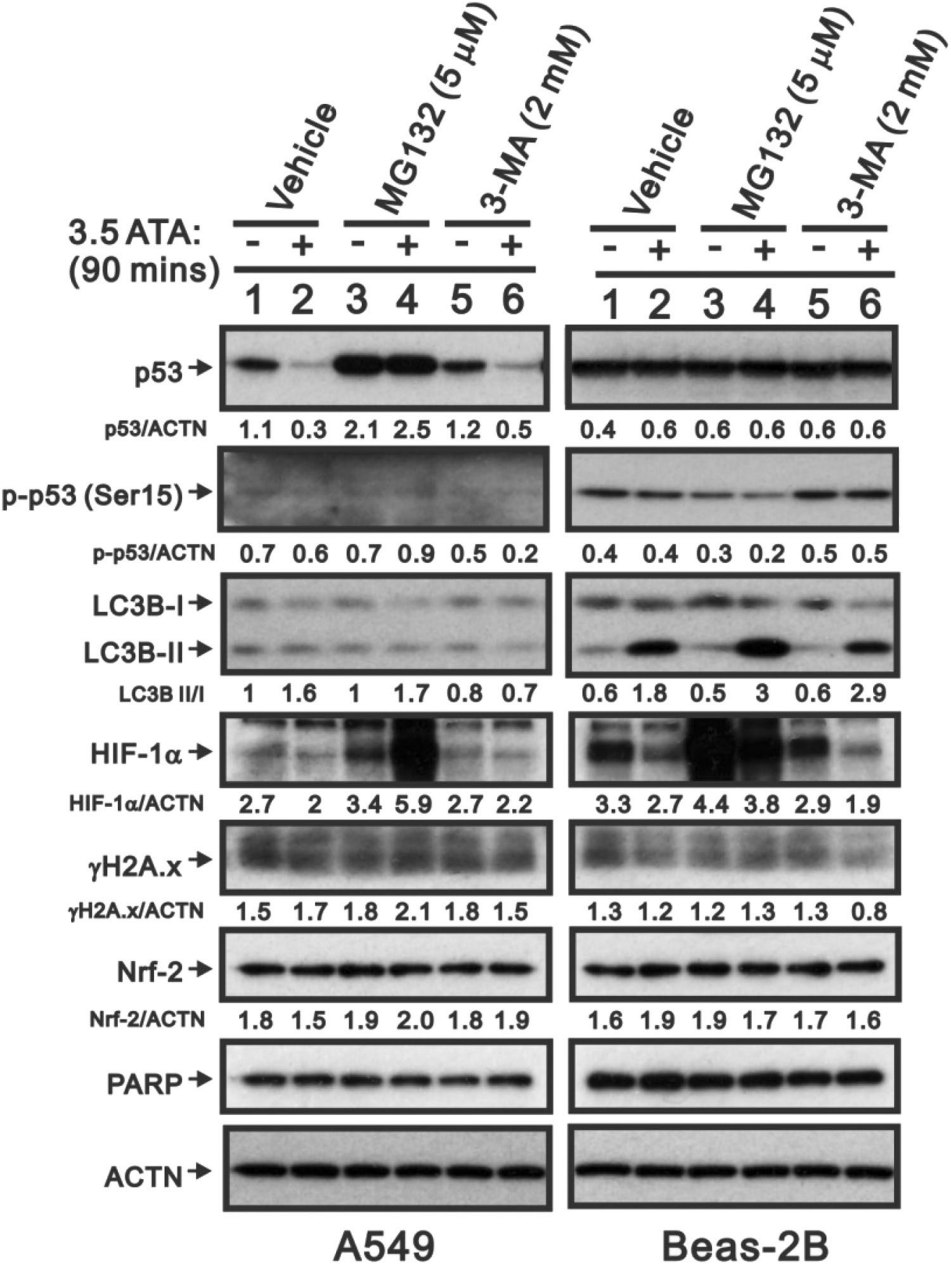
The reduced p53 protein level by HBOT was modulated through a ubiquitin-dependent proteasome degradation in A549 cells. The HBOT A549 and Beas-2 cell were treated with 3.5 ATA for 90 min in the presence of 5 mM MG132 (a proteasome degradation inhibitor) or 2 mM 3-MA(an autophagy inhibitor) for 4 h. These cell lysates were subject to the Western analysis for antibodies against p53, LC3B, HIF-1α, γH2A.x, Nrf2, PARP, and control protein ACTN. The results are representative of two independent experiments. Quantitative analysis of the western blot was listed under each strip.

The downregulation of p53 protein of A549 cells upon HBOT was consistent at a continuous 3-day exposure; p53 protein was immediately downregulated at the first moment of HBOT exposure and back to normal after 20 h exposure (Fig. 6). Hypoxia-inducible factor 1 alpha (HIF-1α) protein expression was significantly decreased by HBOT after exposure at the first interval and rebounded to basal level both in A549 and Beas-2B cells (Fig. 6); however, the apoptotic biomarkers of cleaved PARP and caspase 3 remained unchanged in both A549 and Beas-2B cells (Fig. 6, Figure S1). With the cytosolic and nuclear fractions, we found that the effect of HBOT on p53 proteins occurred in the cytosol and nucleus of A549 cells (data not shown). We tested whether a proteasome degradation inhibitor, MG132 or the autophagy inhibitor 3-methyladenine (3-MA), could rescue the effect of HBOT on p53 and HIF-1α proteins. Our data showed that MG132 could rescue p53 and HIF-1α proteins in A549-HBOT cells (Fig. 7). The effect of MG132 on HIF-1α protein was dependent on cell-context and HBOT. In A549 cells, MG132 stabilized basal HIF-1α protein and further enhanced HIF-1α protein in A549-HBOT cells. However, the HIF-1α protein in MG132-treated Beas-2 cells was still decreased under HBOT condition. 3-MA could suppress the induction of autophagy by HBOT in Beas-2B cells, suggesting that the autophagy did not affect p53 protein stability in A549 cells by HBOT (Fig. 7).

## Discussion

The clinical application of HBOT to cancer treatment has been debated for decades^19^. Malignancy had been considered the contraindication of HBOT since oxygen at raised pressure might enhance tumor growth^25^. However, more and more studies demonstrated that a combination of HBOT might decrease tumor resistance to chemotherapy and radiotherapy^1,10,12^. Reevaluating the direct effect of HBOT in solid cancer could help us understand the potential role of HBOT in cancer treatment. In this study, HBO was used to treat mice transferred with A549 human lung carcinoma in the manner of 2.5 ATA/90 min daily for 2 weeks after 45 days of tumor establishment. HBOT in a cycle of 14 and 28 days both showed significant improvement for tumor hypoxia environment and suppressed tumor growth compared with control mice. Interestingly, tumor vascularity detected by the expressions of CD31 was significantly increased after 14 and 28 days of HBOT; however, VEGF expression did not change as measured in semi-quantitative I.H.C. staining analysis. This finding indicated that the improvement of cancer hypoxia and cancer vascularity by HBOT did not promote cancer growth. For tumor angiogenesis, the new blood vessels are formed when the basement membrane of existing blood vessels is broken and while the vascular basement membrane has been breached, endothelial cells will be divided to form vessels and start to grow. VEGF appears to be the first to induce endothelial cell mitosis in tumor angiogenesis^26,27^. In contrast, CD31, which is a mitogenic factor in wound healing, reacts more sensitive to oxygen tension^23,28^. HBOT significantly increased the expression of CD31 after 14 days of HBOT. Meanwhile, the volume of A549 tumor cells was not growing as increasing expressions of CD31. To evaluate the direct effect of HBOT in A549 cells, the decrease of p53 and HIF-1α proteins was involved in the ubiquitin-dependent proteasome degradation pathway via the proteasome degradation inhibitor MG132 in A549 cells. The extra effect of MG132 on p53 and HIF-1α proteins were observed by HBOT in A549 cells. In a normal lung epithelial cell line, we also observed the decrease of HIF-1α protein with the HBOT, even in the presence of MG132. The involvement of autophagy was verified via the increasing LC3BII/I ratio and its specific inhibitor, 3-MA, in Beas-2 cells, but not in A549 cells. Our findings suggest that HBOT induced tumor suppression in A549-tumor transferred SCID mice might be through modification of tumor microenvironment rather than induction of autophagy in tumor cells themselves.

Malignant tumors have been recognized as a relative contraindication to HBOT_._ It was concerned that HBOT might have cancer growth-enhancing effects. Our findings indicated that the improvement of tumor hypoxia and tumor vascularity by HBOT did not promote tumor growth but inhibit tumor development, which provided evidence for the potential use of HBOT on tumor malignancy. Although the mechanism of HBOT on tumor suppression is not clear, our experiments argue that HBOT results in tumor apoptosis. Moreover, HBOT can increase the formation of reactive oxygen species (ROS) and induce apoptosis of tumor cells^29,30^. In our previous experiments, primary cells and transformed tumor cell lines were treated with either 2.5 ATA or 3.5ATA of 100% oxygen for 6 h, and a significant percentage of apoptosis was compared. HBO-induced apoptosis of cancer cells was through the intracellular accumulation of H_2_O_2_ and O_2_^**.-**^ as well as the involvement of phosphorylation of p38 MAPK^17^. In the present study, non-hematopoietic A549-transferred SCID mice were treated with HBO2.5 ATA/90 min daily for 2 weeks and immunoassaying of cleaved-caspase-3 was demonstrated after 14 and 28 days of HBOT compared with control mice. However, apoptotic biomarkers have no significant difference, including cleaved PARP and caspase 3, directly in A549 cells after HBOT. Our current study is consistent with our previous reports that different origins of different cancer cells result in different threshold to HBOT-induced cancer apoptosis. The possible proposal of tumor suppression in HBOT treated murine xenograft model is that HBOT may provide better tissue vascluogenesis rather than angiogenesis to recruit more inflammatory cells in tumor microenvironment. However, the detailed working mechanisms, such as the vessel length density and detection of markers of endothelial precursor cells, or angioblasts for vascluogenesis, should be addressed in the future studies.

Lung cancers usually start in the cells lining the bronchi, bronchioles or alveoli. Lung cancer development involves multiple genetic abnormalities leading to malignant transformation of the bronchial epithelial cells, followed by invasion and metastasis^31^. One of the most common changes is mutations of the p53 tumor suppressor gene^32–35^. It was found in 45%–70% of adenocarcinomas and 60%–80% of squamous-cell carcinoma. The frequent loss of heterozygosity (LOH) of p53 on chromosome 17p13 suggests p53 is likely involved in the pathogenesis of NSCLC^36,37^. p53 functions as a crucial normal genome protector, located in the cytoplasm and translocates to the nucleus upon cellular stress. Wild type p53 translocates to the mitochondrial surface and directly binds to Bcl-2 family proteins, leading to apoptosis. Furthermore, wild type p53 induces senescence in neighboring tumor cells, inhibits angiogenesis, polarizes macrophages to the anti-tumor M1 phenotype, and activates NK and T cells to trigger immune clearance^38,39^. Herein, our study demonstrated that HBOT immediately downregulated the endogenous p53 protein, and rebounded to the basal level after 20 h of treatment; which could provide the help to improve tumor vascularity, but could not directly cause further apoptosis and cell cycle arrest in the cell base analysis. We are the first to examine the effect of HBOT on wild type p53 in A549 cells. However, the detailed regulatory network of HBOT will be addressed in future studies.

Other interesting findings were the levels of CD31 and VEGF expressions upon HBOT. There was no significant change of VEGF expressions after HBOT supporting our hypothesis that HBOT could not further accelerate tumor angiogenesis and lead to tumor metastasis. By contrast, CD31, Platelet endothelial cell adhesion molecule (PECAM-1) was significantly upregulated after HBOT; which might further recruit more immune cells to the microenvironment for immune clearance^40,41^. Furthermore, a combination of HBOT and either chemotherapy or radiotherapy may provide better treatment in cancer therapy. In addition to p53 protein, HBOT could decrease the HIF-1α protein in A549 and Beas-2 cells, which supported the hypothesis that HBOT could ameliorate the hypoxic condition in cancer cells^42,43^. Combined with our in vivo and in vitro findings suggest that tumor microenvironment interacting with tumor cells is significantly impacted after HBOT in lung cancer.

## Conclusions

Our results demonstrated that HBOT could improve tumor vascularity, tumor hypoxia and potentially target apoptosis-related genes leading to tumor suppression in A549-transferred SCID mice. Our present work may provide direct beneficial evidence for HBOT in the tumor xenograft mouse model and lighten a hope to other solid tumor cancer therapy in the future.

## Supplementary Information

Supplementary Information.
