# Supplementary material for: Hyperbaric oxygen suppressed tumor progression through the improvement of tumor hypoxia and induction of tumor apoptosis in A549-cell-transferred lung cancer
Source: Sci Rep. 2021 Jun 8;11:12033. doi: 10.1038/s41598-021-91454-2 (PMC8187442; doi:10.1038/s41598-021-91454-2)
Supplement: Supplementary file 1 — Supplementary Information. [file 41598_2021_91454_MOESM1_ESM.pdf]

Figure S1

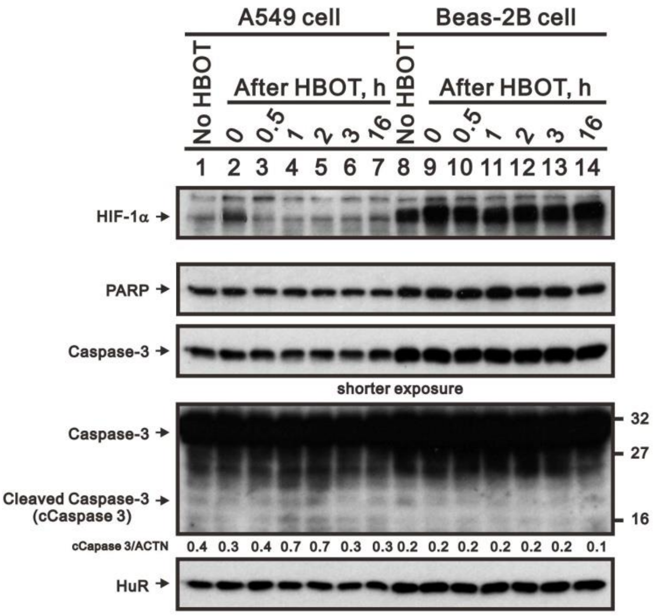

The A549 and Beas-2 cells were treated with HBOT at 3.5 ATA for 90 mins and then lysates were subject to the Western analysis for antibodies against p53, LC3B, HIF-1α, PARP, Caspase 3, and control protein ACTN. The results were representative of two independent experiments. Quantitative analysis of the western blot was listed under each strip.

Supplementary figure S1

Molecular weight for all the following figures (from top to bottom: 180, 135, 100, 75, 63, 48, 35, 25, 17 kDa)

caspase 3 shorter exposure (Lanes 1-7: A549 cell and Lanes 8-14: Beas-2 cell)

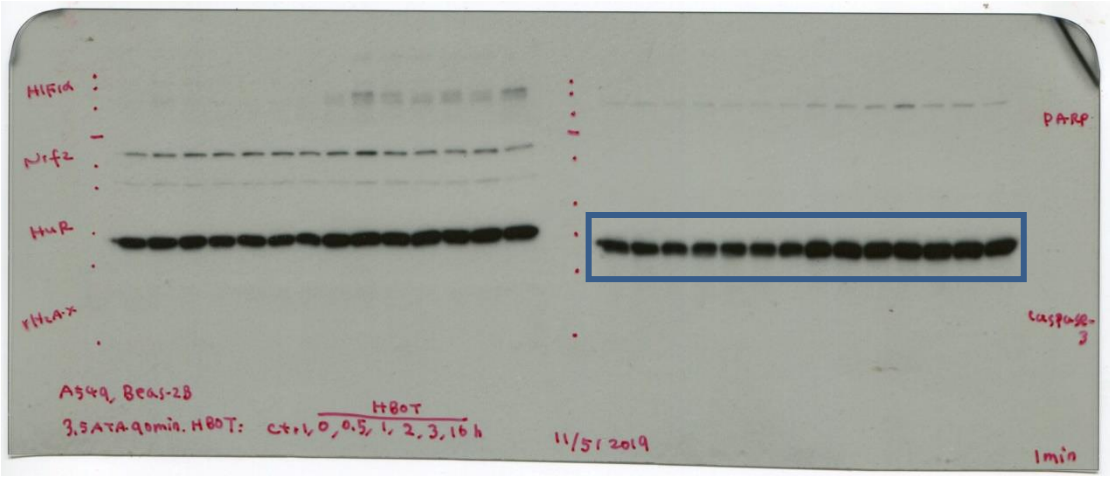

HIF-1 alpha (Lanes 1-7: A549 cell and Lanes 8-14: Beas-2 cell), PARP (Lanes 1-7: A549 cell and Lanes 8-14: Beas-2 cell), Caspase 3 (Lanes 1-7: A549 cell and Lanes 8-14: Beas-2 cell)

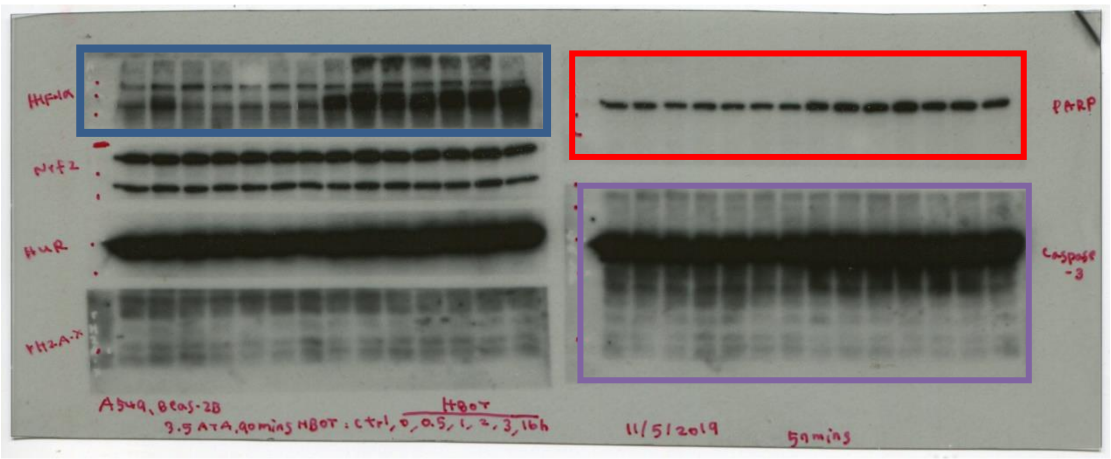

HuR (Lanes 1-7: A549 cell and Lanes 8-14: Beas-2 cell)

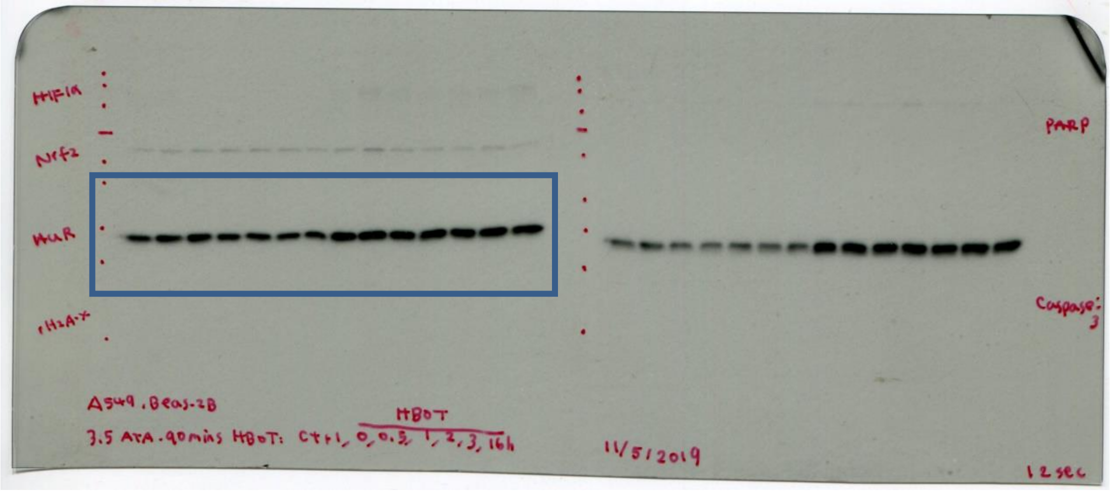

Figure S2

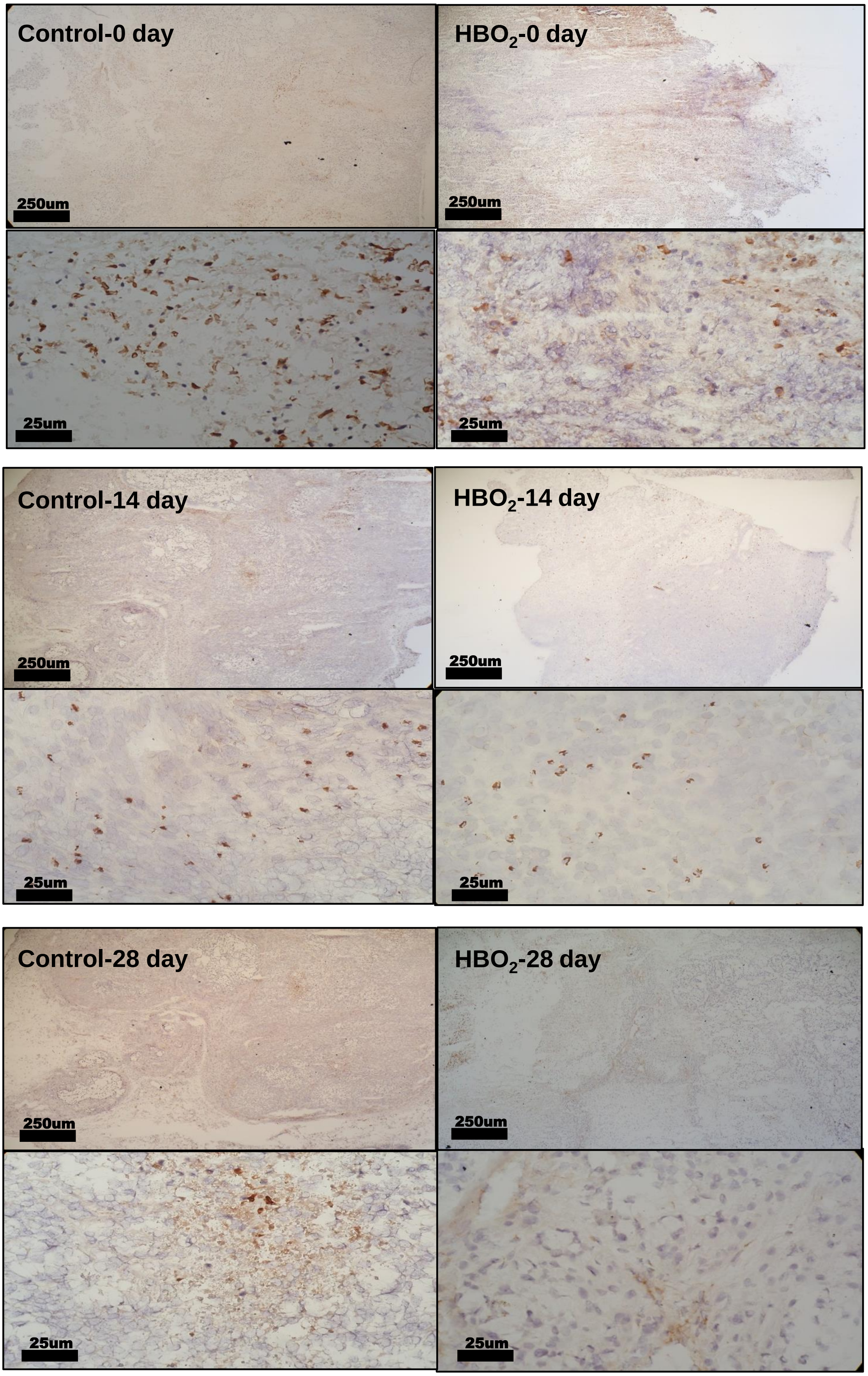

Control-0 day
HBO$_2$-0 day
250um
250um
25um
25um
Control-14 day
HBO$_2$-14 day
250um
250um
25um
25um
Control-28 day
HBO$_2$-28 day
250um
250um
25um
25um

Figure S2. Comparative analysis of immunohistochemical VEGF antigen expressions from groups of mice. No statistical significance of VEGF was observed after 14 days and 28 days of HBOT.

Figure S3

(A)

	Control-0 (N=10)	HBO_2-0 (N=10)	Control-14 (N=10)	HBO_2-14 (N=10)	Control-28 (N=10)	HBO_2-28 (N=10)
Tumor hypoxia	94.0±7.4	92.0±3.1	120.0±9.8	*56.0±12.3	130.0±17.6	*63.0±13.4
CD31	34.0±4.3	31.0±3.8	38.0±3.5	*58.0±5.3	35.0±3.4	*52.0±4.2
VEGFR	22.0±3.6	24.0±3.1	24.0±4.6	23.0±2.2	21.0±5.5	23.0±2.3

*P<0.05, compared with control group

(B)

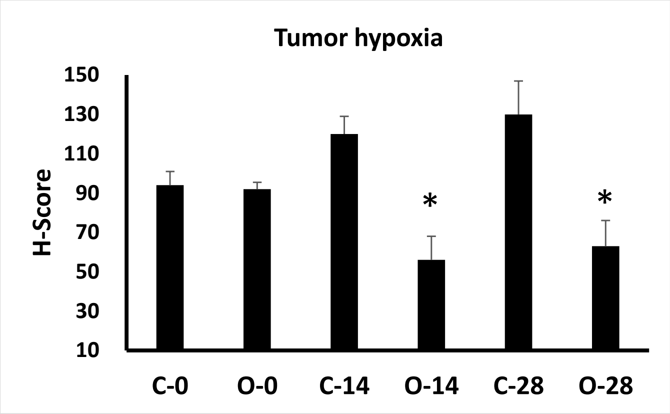

(C)

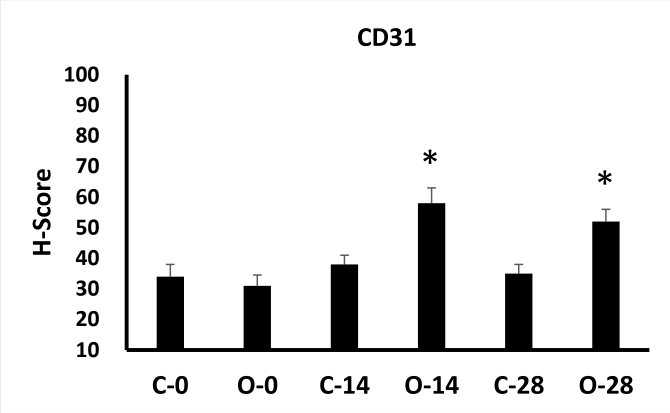

(D)

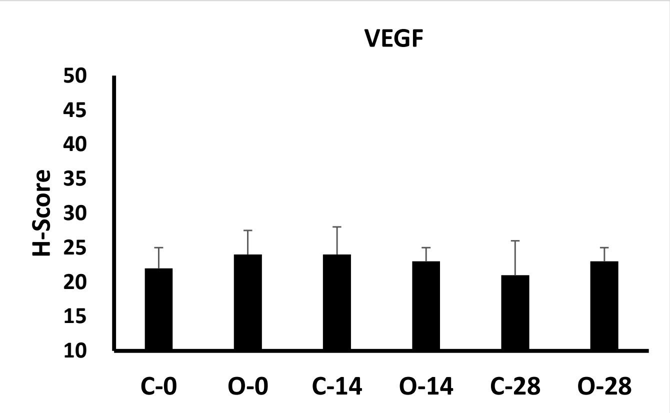

Figure S3. Quantitative expressions of tumor hypoxia, CD31 and VEGF in groups of mice after 14 and 28 days of HBOT.

*P<0.05, compared with Control group

H-score was defined by immunointensity multiply by staining percentage (range from 0 to 300). Computer counting software of CellQuant and PatternQuant were used (3DHISTECH, Budapest, Hungary) for quantitation.

Table S1 Antibodies were used in this study

**Santa Cruz:**		
p53 (DO-1)	sc-126	1:1000
ACTN (H-2)	sc-17829	1:5000
Nrf-2 (A-10)	sc-365949	1:1000
**Abcam:**		
γH2A.x	ab81299	1:1000
**Cell signaling:**		
LC3B	#2775	1:1000
HIF-1α	#14179	1:1000
PARP	#9546	1:1000
Caspase-3	#9662	1:1000
p-p53 (Ser15)	#9286	1:1000
